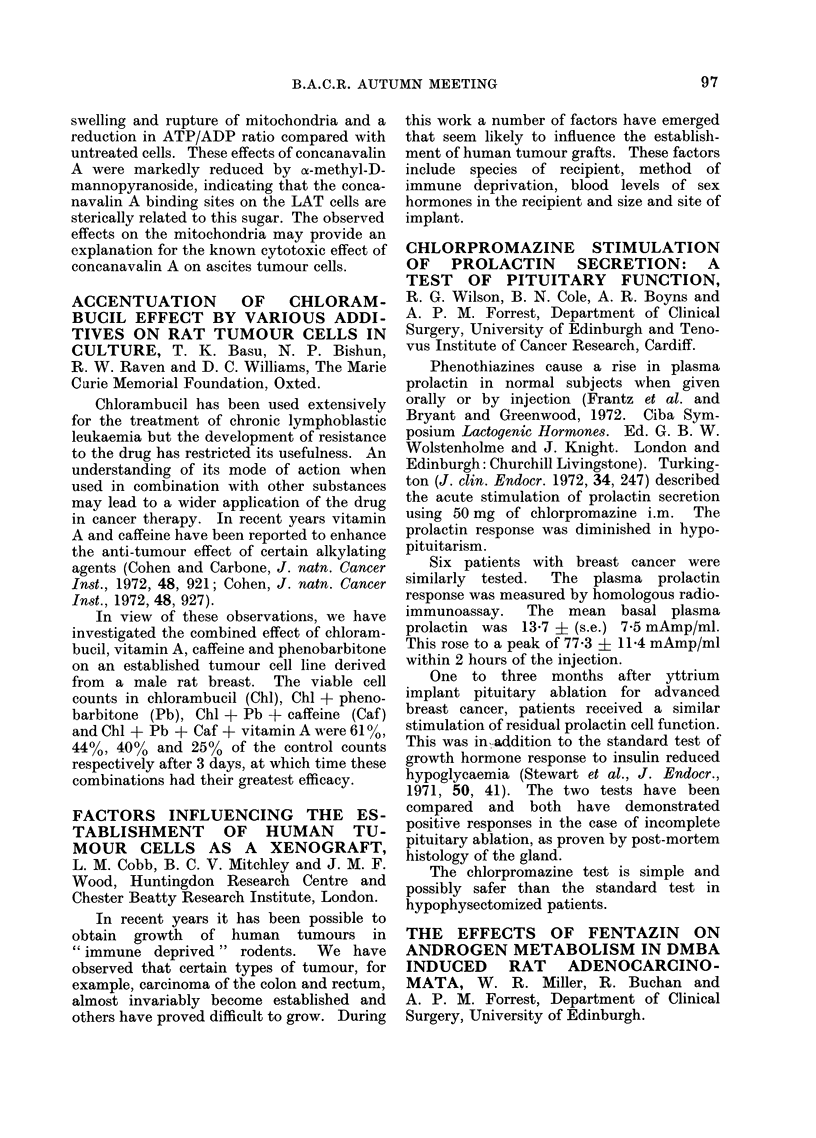# Proceedings: Accentuation of chlorambucil effect by various additives on rat tumour cells in culture.

**DOI:** 10.1038/bjc.1974.30

**Published:** 1974-01

**Authors:** T. K. Basu, N. P. Bishun, R. W. Raven, D. C. Williams


**ACCENTUATION OF CHLORAMBUCIL EFFECT BY VARIOUS ADDITIVES ON RAT TUMOUR CELLS IN CULTURE,** T. K. Basu, N. P. Bishun, R. W. Raven and D. C. Williams, The Marie Curie Memorial Foundation, Oxted.

Chlorambucil has been used extensively for the treatment of chronic lymphoblastic leukaemia but the development of resistance to the drug has restricted its usefulness. An understanding of its mode of action when used in combination with other substances may lead to a wider application of the drug in cancer therapy. In recent years vitamin A and caffeine have been reported to enhance the anti-tumour effect of certain alkylating agents (Cohen and Carbone, *J. natn. Cancer Inst.*, 1972, **48**, 921; Cohen, *J. natn. Cancer Inst.*, 1972, **48**, 927).

In view of these observations, we have investigated the combined effect of chlorambucil, vitamin A, caffeine and phenobarbitone on an established tumour cell line derived from a male rat breast. The viable cell counts in chlorambucil (Chl), Chl + phenobarbitone (Pb), Chl + Pb + caffeine (Caf) and Chl + Pb + Caf + vitamin A were 61%, 44%, 40% and 25% of the control counts respectively after 3 days, at which time these combinations had their greatest efficacy.